# Compressed Lateral and anteroposterior Anatomical Systematic Sequences «CLASS»: compressed MRI sequences with assessed anatomical femoral and tibial ACL's footprints, a feasibility study

**DOI:** 10.1186/s40634-022-00445-3

**Published:** 2022-01-12

**Authors:** Grégoire Thürig, Raùl Panadero-Morales, Luca Giovannelli, Franziska Kocher, José Luis Peris, Moritz Tannast, Daniel Petek

**Affiliations:** 1 Department of Orthopaedic Surgery and Traumatology, Hospital and University of Fribourg, CH-1700 Fribourg, Switzerland; 2 Biomechanics Institute of Valencia, Universitat Politècnica de València, Camino de Vera, 46022 Valencia, Spain; 3 Healthcare Technology Group;Networking Biomedical Research Centre in Bioengineering, Biomaterials and Nanomedicine; Biomechanics Institute of Valencia, Universitat Politècnica de València, Camino de Vera, 4602 Valencia, Spain

**Keywords:** Knee, ACL footprints, MRI

## Abstract

**Purpose:**

This study's main objective is to assess the feasibility of processing the MRI information with identified ACL-footprints into 2D-images similar to a conventional anteroposterior and lateral X-Ray image of the knee. The secondary aim is to conduct specific measurements to assess the reliability and reproducibility. This study is a proof of concept of this technique.

**Methods:**

Five anonymised MRIs of a right knee were analysed. A orthopaedic knee surgeon performed the footprints identification. An ad-hoc software allowed a volumetric 3D image projection on a 2D anteroposterior and lateral view. The previously defined anatomical femoral and tibial footprints were precisely identified on these views. Several parameters were measured (e.g. coronal and sagittal ratio of tibial footprint, sagittal ratio of femoral footprint, femoral intercondylar notch roof angle, proximal tibial slope and others). The intraclass correlation coefficient (ICCs), including 95% confidence intervals (CIs), has been calculated to assess intraobserver reproducibility and interobserver reliability.

**Results:**

Five MRI scans of a right knee have been assessed (three females, two males, mean age of 30.8 years old). Five 2D-"CLASS" have been created. The measured parameters showed a "*substantial"* to "*almost perfect"* reproducibility and an "*almost perfect"* reliability.

**Conclusion:**

This study confirmed the possibility of generating "CLASS" with the localised centroid of the femoral and tibial ACL footprints from a 3D volumetric model. "CLASS" also showed that these footprints were easily identified on standard anteroposterior and lateral X-Ray views of the same patient, thus allowing an individual identification of the anatomical femoral and tibial ACL's footprints.

**Level of evidence:**

Level IV diagnostic study

## Introduction

Anterior cruciate ligament (ACL) lesion incidence is about 0,8 per 100′000 people [7, 16]. ACL reconstruction does not prevent the early onset of osteoarthritis in the long term. Still, it can improve knee kinematic and can reduce the risk of secondary injury to cartilage and meniscus [8]. The outcome of ACL-reconstruction is dependent on careful selection of footprints [4, 10, 25]. The current recommendations tend to recreate the anatomic than isometric footprints [27]. Different options have been described in the literature for proper assessment of femoral and tibial ACL-footprint [12, 19]. Three-dimensional MRI studies showed its reliability [3, 11, 21, 24, 26]. Research has shown that ACL footprints may present a variable location in different individuals [22], and therefore, their intraoperative identification can be challenging. Intraoperative fluoroscopy can help to be more accurate to confirm a proper tunnel placement [14, 20]. However, fluoroscopy alone does not incorporate the footprints' individual anatomical variability. A 2D pre-operative (anteroposterior and lateral) construct showing the individual footprints would be needed and act as a model during surgery and fluoroscopic verification of optimal tunnel placement to fill this lack of information. As most patients who suffer from an ACL tear undergo an MRI scan, we intended to use this image acquisition to create a specific 2D model. This study's main objective is (1) to assess the feasibility of converting MRI information of the ACL-footprints into a 2D image similar to a conventional anteroposterior and lateral X-Ray image of the knee. The secondary aim is (2) to perform specific morphometric measurements. This study is a proof of concept of the technique.

## Material & methods

Five anonymised MRIs of a right knee were analyzed. None of those showed meniscal, cartilage or ligamentous lesion. Patients had no history of fracture or previous surgery of the knee joint. The growth plates were closed, and there was no skeletal dysplasia or osteoarthritis.

### Compressed Lateral and Anteroposterior Anatomical Systematic Sequence ("CLASS")

The same standard MR-technique using an *Optima MR360 1.5 T Advance scan, GE Healthcare* was applied in all cases. All radiographic images were digitally acquired using a picture archiving and communication system (PACS, GE Healthcare, Belgium). The sequences included sagittal proton density fat saturated isotropic 3D with isovoxel of 0,6 × 0,6 × 0,6 mm.

**Fig. 1:**
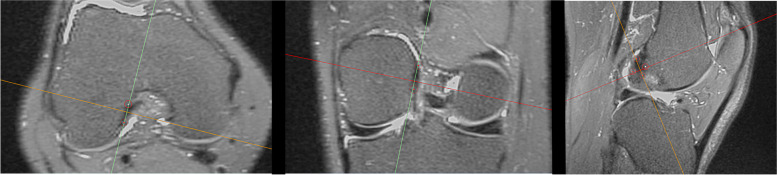
Identification femoral ACL footprint in Materialise Mimics

**Fig. 2:**
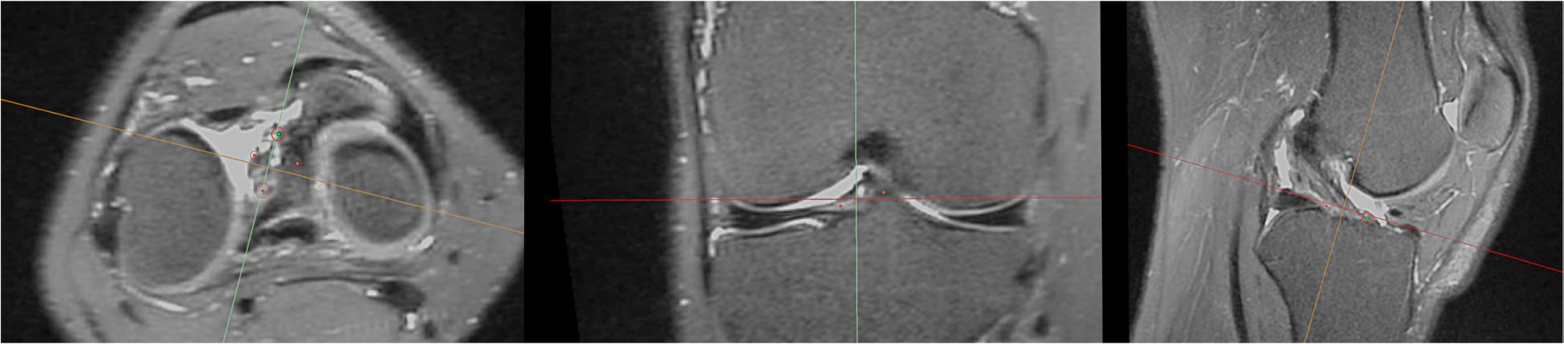
Identification tibial ACL footprint in Materialise Mimics

A senior orthopaedic knee surgeon performed the footprint identification using the *multiplanar reformation* tool of the software *Materialise Mimics® 17.0 research*. First, the femoral ACL’s footprint was identified in the axial view to match the sagittal orientation towards the lateral wall of the notch. The sagittal orientation was then matched to it in the coronal view as well. Finally, the coronal orientation was aligned with the fibers of the ACL in the sagittal view. The femoral footprint was then marked from four points as follow: shallow, deep, high and low (Fig. 1). To identify the tibial ACL’s footprint, the sagittal orientation was kept aligned with the fibers of the ACL. The axial orientation was then matched to the tibial joint line in the coronal view. Finally, the axial orientation was aligned with the tibial slope between the tibial spines. The tibial footprint was then identified from four points as follow: anterior, posterior, medial and lateral (Fig. 2). All points were selected at their maximal margins. The previously mapped four points were computed by coordinate averaging with an in-house routine GNU Octave (version 4.0.0) to generate a centroid femoral and tibial optimised footprint. The fibular head's styloid process acting as a reference was identified with a single point. The in-house GNU Octave scripts computed the centroid points' position on the 2D-image reference system and the pixel intensity by averaging the pixel intensity values along the projection direction. By projecting the MRI volumetric image and the calculated points on the lateral and anteroposterior views, the "CLASS" MRI sequence was established.

### Radiographic evaluation

**Fig. 3:**
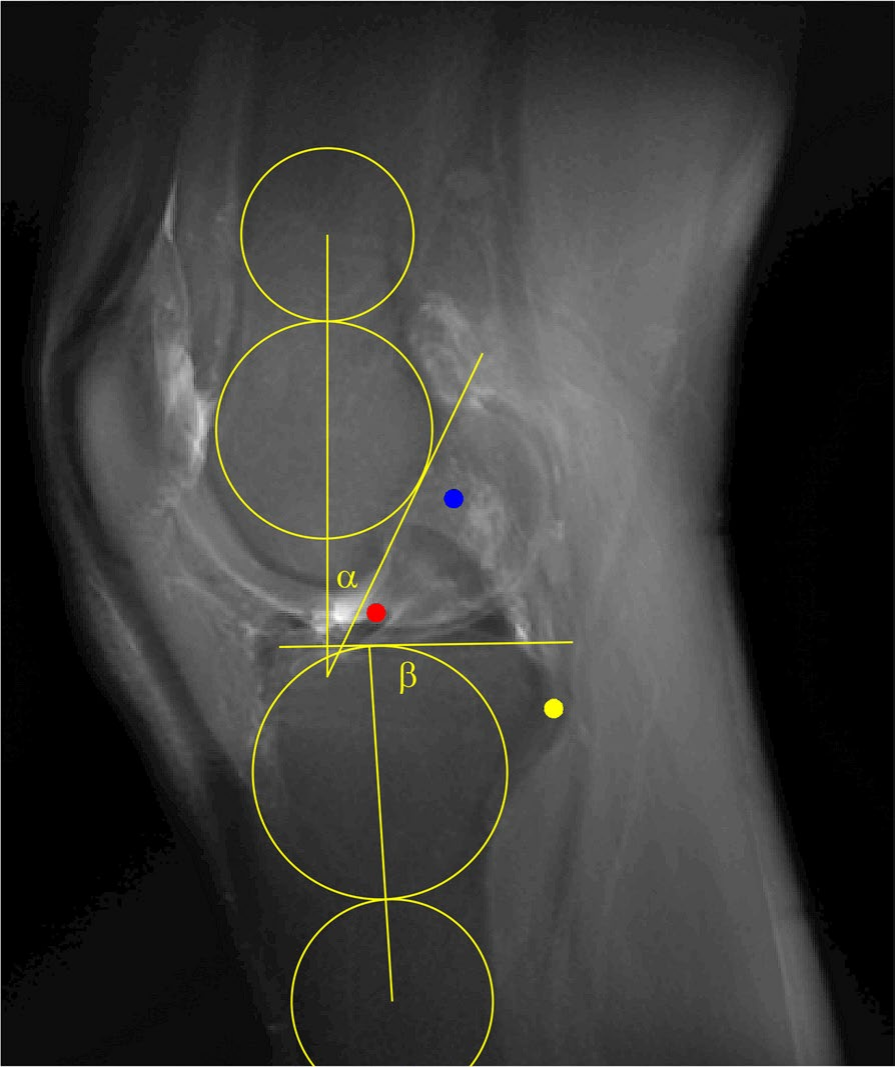
femoral intercondylar notch roof angle ("α"), proximal tibial slope ("β")

Using the software ImageJ, several measurements have been performed on the CLASS [23]. On the anteroposterior (AP) view, the location of the ACL tibial footprint was established ("coronal ratio tibial footprint"). The sagittal location of the ACL tibial footprint was determined by applying the reference line described by Amis and Jakob [1] ("sagittal ratio tibial footprint") on the lateral (LAT) view. The sagittal location of the ACL femoral footprint was determined by using the reference line described by Amis and Zavras [2] ("sagittal ratio femoral footprint high to low" and "sagittal ratio femoral footprint deep to shallow") on the lateral view as well. The femoral intercondylar notch roof angle ("α") on the lateral sequence was determined using the longitudinal axis of the femur and the Blumensaat line. Two circles were drawn, one tangent to the Blumensaat line and the anterior and posterior femur edges and the other tangent to the proximal border of the distal circle, anterior and posterior femur edges. The longitudinal femoral axis was then assessed by connecting the centres of both circles (Fig. 3). The proximal tibial slope ("β") was measured using the longitudinal tibial axis according to Lipp [15] and the articular surface (Fig. 3). The angle between the tibial articular surface and the ACL footprints was calculated from both the anteroposterior and lateral images ("coronal articular surface and ACL—angle" and "sagittal articular surface and ACL—angle"). On the lateral sequence, the angle between the tibial articular surface and the Blumensaat's line was measured ("sagittal articular surface and Blumensaat's line—angle"). The image analysis and angles measurements have been performed independently from each other at two different time frames with a 3-weeks interval. Patients' names and identifying features were blinded to minimise recall bias. To assess the intraobserver reproducibility and interobserver reliability, intraclass correlation coefficient (ICCs) was calculated including 95% confidence intervals (CIs) based on a mean-rating (*k* = 2), absolute agreement, 2-way mixed-effects model. The ICC was graded as ICC < 0.20 for *slight*; 0.21 to 0.40 for *fair*; 0.41 to 0.60 for *moderate*; 0.61 to 0.80 for *substantial*; and > 0.80 for *almost perfect* agreement [17]. Descriptive analysis has been performed using IBM SPSS Version 26.0 (SPSS Inc, Chicago, IL). The study was carried out following the World Medical Association Declaration of Helsinki. According to federal law, our project did not need the approval of the local ethical committee (http://www.cer-vd.ch/soumission/premiers-pas.html).

## Results

**Fig. 4:**
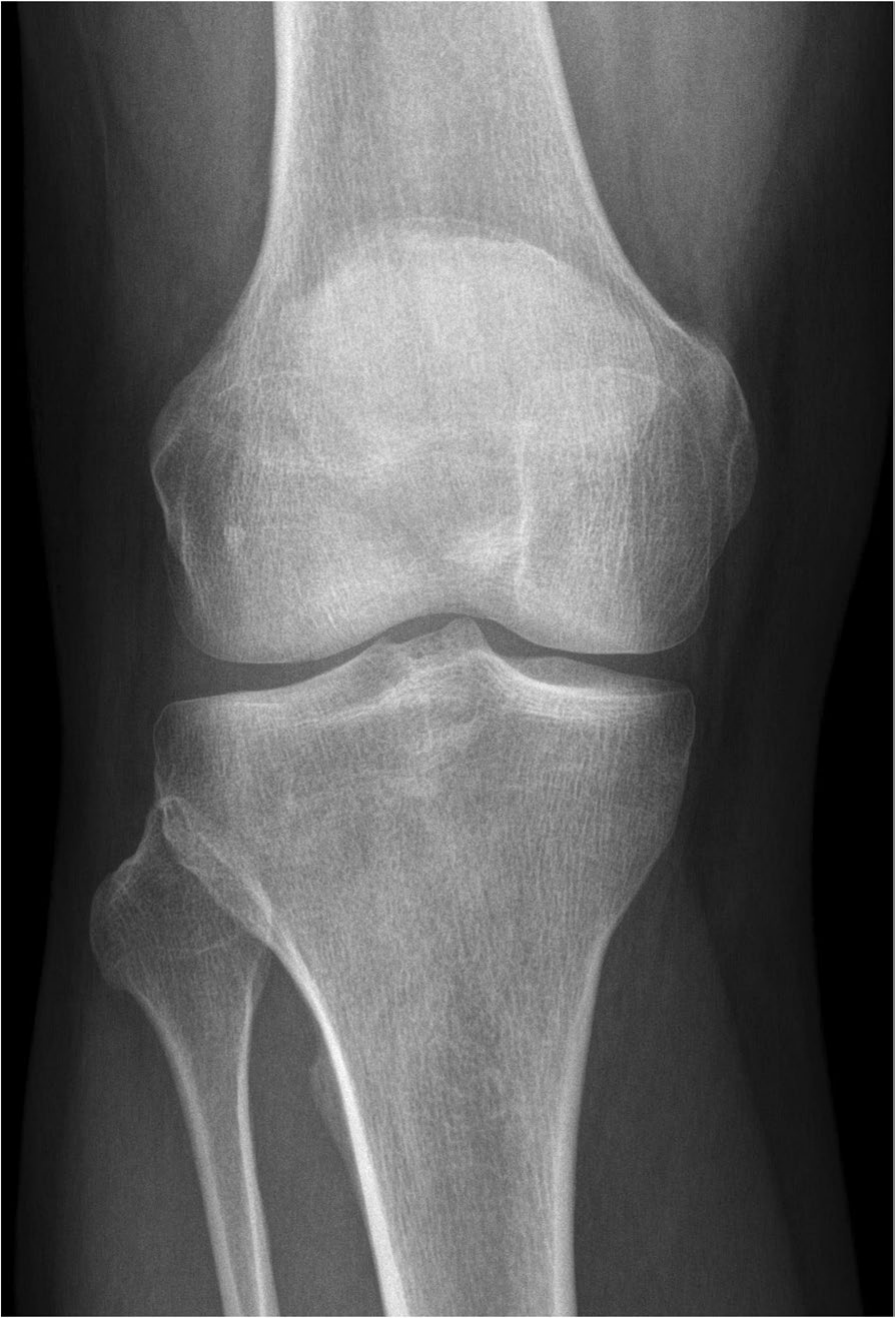
AP conventional X-ray

**Fig. 5:**
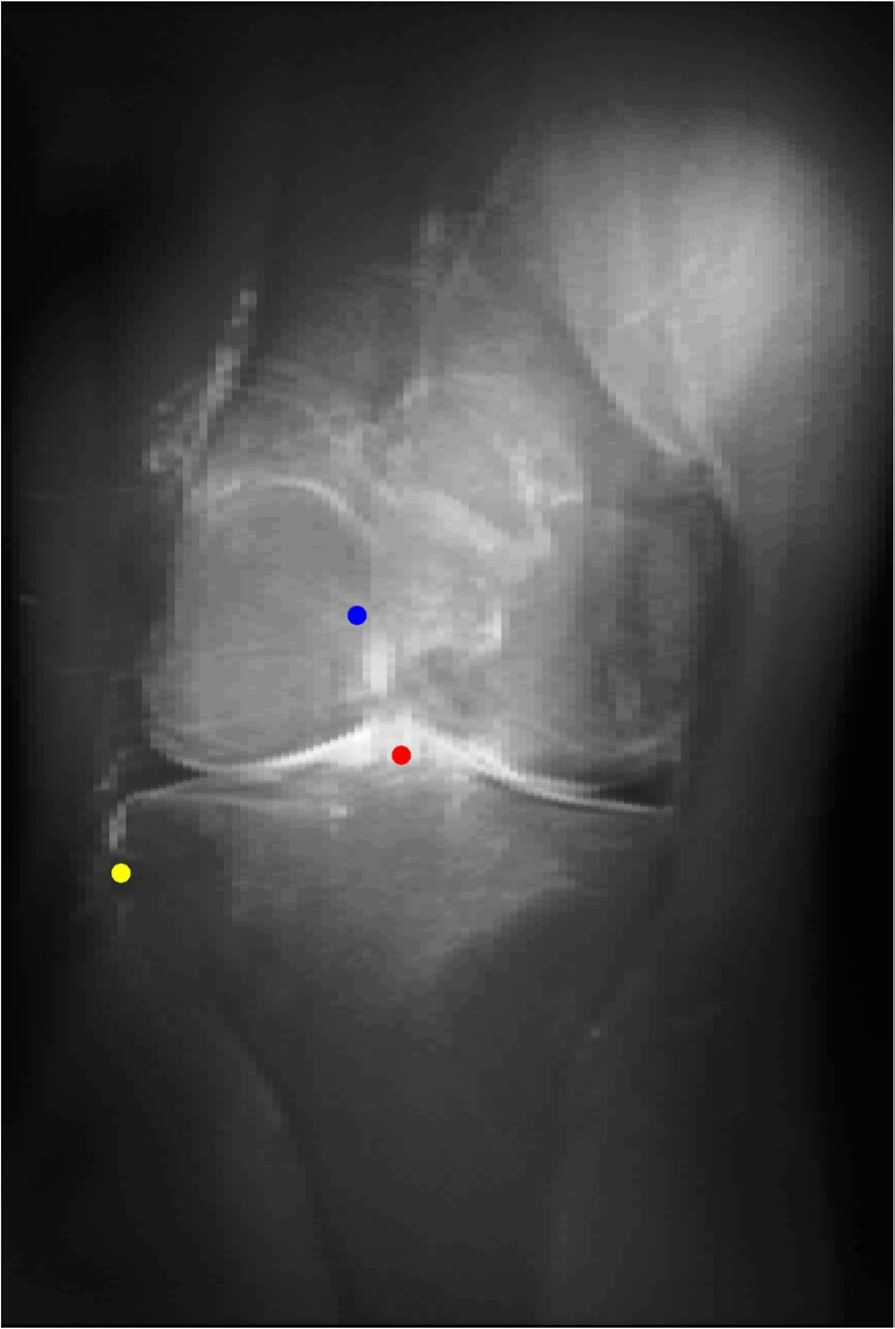
AP "CLASS" view (blue point: centroid ACL femoral footprint, red point: centroid ACL tibial footprint, yellow point: the styloid process of fibular head)

**Fig. 6:**
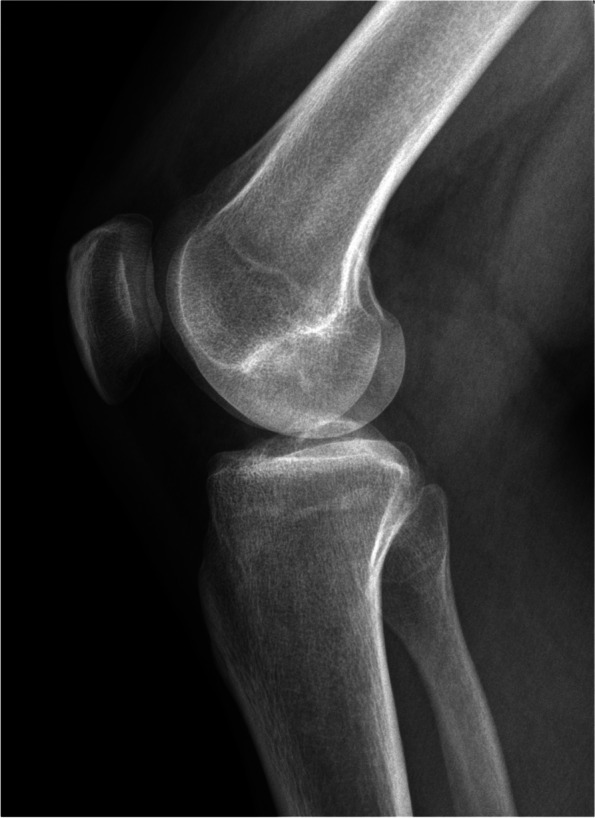
LAT conventional X-ray

**Fig. 7:**
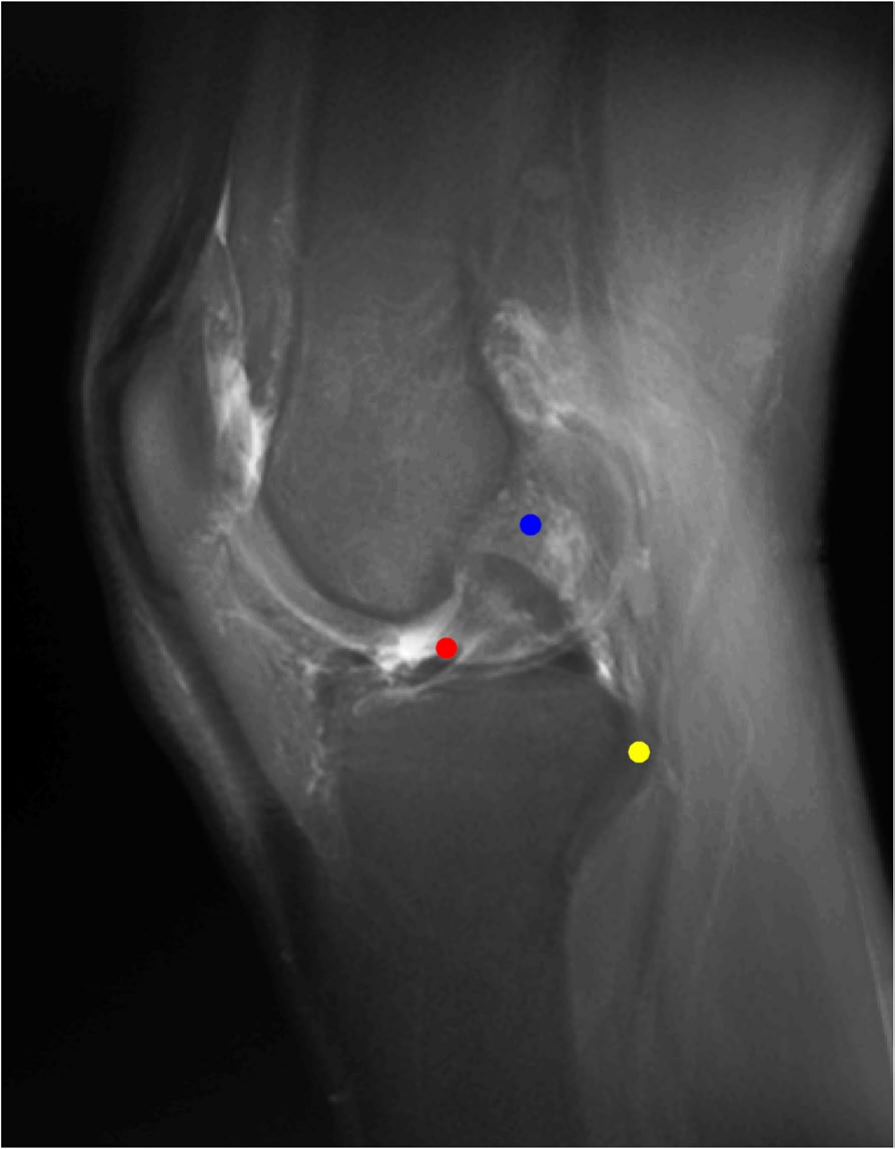
LAT "CLASS" view (blue point: centroid ACL femoral footprint, red point: centroid ACL tibial footprint, yellow point: the styloid process of fibular head)

Five MRIs of a right knee have been assessed (three females and two males, mean age of 30.8 years old). The Figs. 4 and 5 show the conventional radiogram of a right knee (AP and LAT views). Fig. 6 and 7 show the 2D-compressed anteroposterior and lateral views. Table 1 shows the results of the measurements mentioned above and performed on the newly acquired "CLASS" views.

**Table 1:** Intra and interobserver measurement results

*N* = 5	Mean	Reproducibility (ICC reader 1)	Reproducibility (ICC reader 2)	Reliability (ICC reader 1 versus reader 2)
Age (years)	30.8 (29 – 33)	n/a	n/a	n/a
coronal ratio tibial footprint	0.506 (0.501 – 0.512)	0.827 with CI 95% (-0.204 – 0.981)	0.831 with CI 95% (-0.133 – 0.983)	0.891 with CI 95% 0.589 – 0.987)
sagittal ratio tibial footprint	0.429 (0.405 – 0.441)	0.816 with CI 95% (-0.213 – 0.980)	0.994 with CI 95% (0.953 – 0.999)	0.953 with CI 95% (0.787 – 0.995)
sagittal ratio femoral footprint high to low	0.222 (0.203 – 0.237)	0.922 with CI 95% (0.180 – 0.992)	0.889 with CI 95% (-0.120 – 0.989)	0.940 with CI 95% (0.662 – 0.993)
sagittal ratio femoral footprint deep to shallow	0.370 (0.36 – 0.38)	0.977 with CI 95% (0.770 – 0.998)	0.985 with CI 95% (0.880 – 0.998)	0.968 with CI 95% (0.850 – 0.996)
α	34,243° (31.488 – 35.393)	0.934 with CI 95% (0.005 – 0.994)	0.991 with CI 95% (0.935 – 0.999)	0.962 with CI 95% (0.841 – 0.996)
β	86.967° (86.129 – 87.579)	0.922 with CI 95% (0.234 – 0.992)	0.783 with CI 95% (-0.085 – 0.977)	0.879 with CI 95% (0.544 – 0.986)
coronal articular surface and ACL—angle	73.309° (72.635 – 74.048)	0.943 with CI 95% (0.586 – 0.994)	0.936 with CI 95% (0.470 – 0.993)	0.967 with CI 95% (0.873 – 0.996)
sagittal articular surface and ACL—angle	60.253° (59.980 – 60.550)	0.980 with CI 95% (0.827 – 0.998)	0.963 with CI 95% (0.665 – 0.996)	0.986 with CI 95% (0.944 – 0.998)
sagittal articular surface and Blumensaat’s line—angle	58.634° (57.691 – 58.989)	0.990 with CI 95% (0.901 – 0.999)	0.989 with CI 95% (0.855 – 0.999)	0.975 with CI 95% (0.902 – 0.997)

## Discussion

This study's main objective was to evaluate the feasibility to create 2D-images mimicking an anteroposterior and lateral conventional X-ray expressing precisely the location of the tibial and femoral ACL footprints based on a standard MRI acquisition of a knee. Secondary aims consisted of evaluating the possibility of conducting different measurements used in standard knee radiographic analysis. The intrarater reproducibility and reliability were for all measurements *almost perfect,* according to Montgomery [17]. Amis and Zavras [2] presented their review on “isometricity and graft placement during anterior cruciate ligament reconstruction”. Based on the illustrated basic principles of isometry and the results found in the literature, they concluded a “close to isometric” zone to be the preferred choice for femoral tunnel placement, and suggested placement at 20% high to low (HL) and 38% deep to shallow (DS) based on the quadrant method. Bernard and Hertel [5] performed an anatomical footprint analysis on 10 cadaveric knees based on the same method. According to their results, the center of the anatomic femoral ACL insertion was located at 28.5% HL and at 24.8% DS. Piefer J.W. et al. [19] did a systematic review on evaluation of the anatomic femoral ACL-footprint and presented a mean value of 35,2% HL and 28.5% DS. Parkar et al. [18] performed a systematic review regarding anatomic location centers of the femoral and tibial ACL footprints and calculated the weighted median of the ACL femoral insertion center to be 34% and 26% in the HL and DS directions, respectively. The 5th and 95th percentiles were 28% and 43%, respectively, for HL, and 24% and 37%, respectively, for DS. Iriuchishima et al.[13] did a systematic study on the performed methods and tunnel placement strategies in anatomical single-bundle ACL reconstruction. Evaluation of 19 studies showed a targeted femoral footprint center at 32.3 ± 7% HL and at 30.6 ± 4.3% DS. Our results showed a mean centroid femoral footprint at 22.2% HL and 37.0% DS in a young and healthy population. Byrne et al. [6] performed a retrospective study to assess femoral tunnel position on routine postoperative radiographs in patients who required ACL revision compared with patients who did not require revision. In patients who did not require revision, the femoral tunnel was 38% ± 9% HL and 28% ± 6% DS. It was shown that too anterior and too high femoral tunnel placement were independent risk factors for ACL revision surgery. It should be noted that in this study, tunnel position was analyzed using only postoperative radiographs. However, the reasons for failed ACL reconstruction may be multifactorial and should be correlated accordingly, as also emphasized in this publication.

Our results showed a mean centroid femoral footprint at 22.2% HL and 37.0% DS in a young and healthy population, and appears to be closer to the isometric footprint suggested by Amis and Zavras [2] than to the median of the systematic reviews. The wide range in the determined anatomical femoral footprint in the literature suggests that this should be correlated with the tibial footprint and other morphological criteria to gain a better understanding. Amis and Jakob [1] described the anatomical tibial ACL-centre as 43% anteroposterior in the lateral standard X-Ray view. Others authors showed similar results with a range of 40 – 46.2% [9]. This variation suggests a patient dependent anatomical ACL-centers. Our result of 42.9% (40,5 – 44,1%) is comparable for this small series of patients [9]. This method's strength is that the MRI information is not altered to create the 2D-compressed images. The MRI-slices keep most of the MRI information enriched with the ACL femoral and tibial footprints' location. According to the exclusion criteria, all knees did not show any pathologies. There are some limitations to this study. As the sample size was rather small, further studies using these 2D-compressed images with a larger collective are needed. Its major strength is that this sequence may give a better understanding of the anatomical femoral and tibial ACL's footprints. This would allow an individual approach for tunnel placement in ACL reconstruction surgery to assess the anatomical variation of the ACL anatomy among the population. Therefore, it could be used as a template during surgery for tunnel placement under fluoroscopic control, for postoperative evaluation of tunnel placement, or as input data for fluoroscopy-based navigation systems.

## Conclusion

This study confirmed the possibility to compress the information of a 2D/3D-MRI scan of a knee with individually localised tibial and femoral footprints into a "Compressed Lateral and Anteroposterior Anatomical Systematic Sequence". Specific morphometric measurements can be performed using the newly generated sequences, giving the possibility for individual identification of anatomical femoral and tibial ACL's footprints.

## Disclosures

Each author certifies that his institution has approved the reporting of this case series that all investigations were conducted in accordance with the Declaration of Helsinki and Guidelines for Good Clinical Practice.

## Abbreviations

ACL: Anterior cruciate ligament
AP: Anteroposterior
CI: Confidence intervals
CLASS: Compressed Lateral and anteroposterior Anatomical Systematic Sequences
DS: Deep to shallow
HL: High to low
ICC: Intraclass correlation coefficient
LAT: Lateral
α: Femoral intercondylar notch roof angle
β: Proximal tibial slope
